# Vitamin D and Gestational Diabetes Mellitus: Is There a Link?

**DOI:** 10.3390/antiox8110511

**Published:** 2019-10-25

**Authors:** Gianluca Rizzo, Simone Garzon, Michele Fichera, Marco Marzio Panella, Ursula Catena, Antonio Schiattarella, Pasquale de Franciscis, George Vilos, Jan Tesarik, Péter Török, Giuseppe Grosso

**Affiliations:** 1 Independent Researcher; gianlucarizzo@email.it; 2 Department of Obstetrics and Gynecology, “Filippo del Ponte” Hospital, University of Insubria, 21100 Varese, Italy; simone.garzon@yahoo.it; 3 Department of General Surgery and Medical Surgical Specialties, University of Catania, 95124 Catania, Italy; fichera.michele@policlinico.unict.it (M.F.); mpanella@unict.it (M.M.P.); 4 Division of Gynecologic Oncology, Fondazione Policlinico Universitario Agostino Gemelli, IRCCS, 00168 Rome, Italy; ursula.catena@gmail.com; 5 Department of Woman, Child and General and Specialized Surgery, University of Campania “Luigi Vanvitelli”, 81100 Naples, Italy; aschiattarella@gmail.com (A.S.); pasquale.defranciscis@unicampania.it (P.d.F.); 6 Department of Obstetrics and Gynecology, Division of Reproductive Endocrinology and Infertility, Western University, London, ON N6H5W9, Canada; George.Vilos@lhsc.on.ca; 7 MARGen Clinic, 18006 Granada, Spain; jtesarik@clinicamargen.com; 8 Faculty of Medicine, Institute of Obstetrics and Gynaecology, University of Debrecen, H-4032 Debrecen, Hungary; drtorokp@freemail.hu; 9 Department of Biomedical and Biotechnological Sciences, University of Catania, 95123 Catania, Italy

**Keywords:** vitamin D, gestational diabetes mellitus, pregnancy, nutrition, supplements, antenatal care, food fortification

## Abstract

Gestational diabetes mellitus (GDM) is a very common condition among pregnant women worldwide with important metabolic implications on the mother and the offspring. Vitamin D status has been suggested to potentially play a role in GDM risk with no documented consequences for the offspring. The purpose of this article was to review currently available evidence on the relationship between vitamin D and GDM. Current evidence shows an association between vitamin D blood levels and risk of GDM, despite heterogeneity of results across studies limit the conclusions. Moreover, data from randomized controlled trials is scarce and resulting in null findings. Among the limitations to be noted, improving the standardization of dosages, the characteristics of individuals in the sample, and the appropriate outcome measurement could provide a more effective approach in understanding the relationship between vitamin D and GDM. In conclusions, despite observational studies may suggest that adequate vitamin D levels may decrease the risk of GDM compared to deficiency status, evidence from clinical trials is inadequate to draft any definitive conclusion regarding its supplementation. Future better designed randomized clinical trials taking into account a more integrated approach could provide clearer and definitive data on the outcomes of such a multifactorial condition.

## 1. Introduction

Interest in vitamin D has increased in the last decades with the improvement of the knowledge regarding its wide implication in several physiological processes. In fact, although its involvement in bone metabolism and calcium management in bone health processes is widely confirmed, literature repeatedly suggested non-musculoskeletal targets such as the immune system, regulation of cell proliferation and differentiation, and glucose metabolism [1,2,3,4]. Interestingly, recent evidence demonstrated that vitamin D may also influence metabolic parameters in both menopausal and postmenopausal women [5,6], and, consequently, being potentially associated with human reproduction [7,8,9] and birth outcomes [10].

In pregnancy, external *stimuli* can affect mother-foetus dyad with possible consequences for both the mother and the offspring [11,12]. Thus, nutritional recommendations in this condition may be specific and significantly different from those referring to adult women in general, taking into account that adaptation for relevant nutrients would affect both mother and foetal health [13,14,15,16]. The need of vitamin D could be higher in pregnancy [17]; in fact, many populations, both among developing and industrialized countries, still suffer from vitamin D deficiency in pregnancy with a variable incidence ranging from 40% to 100% [18,19,20,21,22]. Interestingly, vitamin D has been suggested as a potential candidate for prevention of gestational diabetes mellitus (GDM), despite conflicting current opinions [23,24,25]. The purpose of this review is to summarize current evidence on the possible link between vitamin D and GDM, briefly discussing the potential mechanisms underlying this relation and providing data from meta-analysis and systematic reviews.

## 2. Potential Role of Vitamin D on GDM Pathogenesis

Increased blood glucose levels stimulate pancreatic β-cells secretion of insulin. Peripheral tissues respond to this hormone by activating glucose transport inside the cells. The balance between insulin secretion and its peripheral sensitivity represents the modulation system for carbohydrates tolerance [26]. The chronic stimulation of this modulation system by high glycaemic levels and consequent insulin resistance leads to an exhaustion of pancreatic secretion competence that provokes alteration of insulinemia, leading to glucose intolerance and even type-2 diabetes mellitus (T2DM). 

GDM is defined as an alteration of glucose tolerance with variable severity, with the first onset during pregnancy, in the absence of a diagnosis of T2DM [27]. The GDM prevalence ranges from 1% to 25% depending on population reference and diagnostic cut-offs [28,29]. It represents the leading cause of complications for mother and offspring [30]. Moreover, GDM is a determinant for serious long-term complications such as obesity, T2DM, metabolic syndrome for both mother and the offspring, with a risk for the establishment of a trans-generational predisposition [31]. 

Pregnancy is a condition that promotes physiological insulin resistance. Higher maternal glucose levels allow the adequate delivery to the foetus through the placental syncytium. This well-known phenomenon is mediated by steroid hormones and by the anti-insulin action of placental tissues [32], which in turn can modulate insulin signalling, glucose transport and peroxisome proliferator-activated receptor (PPAR) function [33,34], which placenta expression has been directly associated with GDM in female population [35]. However, if there is latent and pre-existing glucose intolerance, the pregnancy can uncover a pancreas inadequacy for compensating the secretion in response to the peripheral insulin resistance, resulting in a β-cells dysfunction through a physio-pathological continuum [36].

Vitamin D can influence glucose homeostasis with multiple mechanisms:

- Functional pancreatic alteration can be associated with immune cell infiltration among glandular cells with consequent inflammation [37]. Vitamin D exerts anti-inflammatory properties that can drive the recovery of physiological insulin secretion [38].
- Insulin receptor of peripheral cells drives receptor-mediated endocytosis routing calmodulin-dependent intracellular signalling [39]. Vitamin D enhances duodenal absorption and renal resorption of calcium that is therefore available to the intracellular signalling activated by insulin [40].
- Interaction between insulin-like growth factor (IGF) and molecular partners of vitamin D pathways could play a role in glucose homeostasis.
- Vitamin D receptors (VDRs) were described in different extra-bones peripheral tissues which explains the wide non-musculoskeletal functions of vitamin D, including the action on insulin receptor that promotes insulin sensitivity [41]. Pancreatic β-cells show VDRs with a possible modulating action of vitamin D on insulin secretion [42,43].
- Vitamin D can act indirectly through the reduction of risk factors common to GDM such as obesity. Vitamin D is a fat-soluble vitamin and its migration from the bloodstream to fat deposits can reduce its availability [44]. Moreover, obese patients have higher levels of vitamin D binding protein (VDBP) that can reduce vitamin D bioactive fractions [45].

## 3. Cut-offs and Methods of Detection

Diagnosis of GDM includes fasting blood glucose concentration and/or oral glucose tolerance test (OGTT), depending on the organization releasing the diagnostic procedure and cut-offs [46,47,48,49]. Specifically, the American College of Obstetricians and Gynaecologists (ACOG) recommendations require two or more threshold values for the diagnosis, while the Canadian Diabetes Association (CDA), the International Association of Diabetes and Pregnancy Study Groups Consensus Panel (IADPSG), and the World Health Organization (WHO) require only one. About cut-offs, fasting plasma glucose ≥ 5.3 mmol/L (95 mg/dL) is adopted by CDA and ACOG, while 5.1 mmol/L (92 mg/dL) is adopted by WHO and IADPSG. Except for ACOG that use 100 g of glucose, all organizations use 75 g of glucose for the OGTT. The 1h plasma glucose cut-off of ≥ 10.0 mmol/L (180 mg/dL) is adopted by all but CDA that uses 10.6 mmol/L (191 mg/dL) value. There is great variability in 2 h plasma glucose cut-offs: ≥ 8.5 (153 mg/dL) for IADPSG and WHO; ≥ 9 (162 mg/dL) for CDA; and ≥ 8.6 (155 mg/dL) for ACOG. A 3 h plasma glucose is adopted only by ACOG with a cut-off of 7.8 mmol/L (140 mg/dL). The American Diabetes Association (ADA) adopted two different strategy for the screening of GDM: A one-step method requiring only one glucose plasma value exceeding fasting, 75 g OGTT at 1 or 2 h with cut-offs equivalent to IADPSG; or a two-step method requiring a first non-fasting 50 g OGTT evaluation, followed by a 100 g OGTT with criteria, and cut-offs equivalent to ACOG. A 2 h 75 g OGTT adoption for GDM diagnosis were confirmed in the international and multicentre HAPO Study, based on 25,505 pregnant women outcomes [50]. 

Even the vitamin D cut-offs are widely debated with no shared consensus, especially for pregnancy. Indeed, it is not clear whether blood levels of vitamin D during pregnancy are stable through all phases of gestation and also if they can be the same for adults in a general population. 

There are some difficulties in choosing the adequate gold standard to set the cut-off for the vitamin D. In the attempt to validate vitamin D blood levels of adequacy, the value above which the parathyroid hormone (PTH) reaches its plateau in response to the vitamin D was chosen [51]. This plateau seems to be reached in adults with 25-hydroxyvitamin D (25(OH)D) blood concentrations ranges from 30 to 40 ng/ml (75–100 nmol/L). However, the reverse correlation between vitamin D and PTH blood levels is not accepted by all researchers [52,53,54,55]. PTH concentration depends on the duration of vitamin D deficiency and on calcium that can mask vitamin D insufficiency [56]. In pregnancy, PTH levels do not seem to correlate with vitamin D [57,58]. However, Kramer at al. highlighted a link between PTH and GDM with the finding that the prevalence of GDM increased while increasing the PTH [59]. 

Cut-offs of vitamin D were also evaluated on calcium absorption that shown an increased intestinal efficiency when postmenopausal women increased their 25(OH)D blood levels from 20 ng/ml (50 nmol/L) to 32 ng/ml (80 nmol/L) [60]. In a review on clinical practice by Holick and co-workers, routine population screening for vitamin D was not recommended with the higher straight of evidence and the maximum quality for the statement [61]. However, pregnancy was considered an “at risk” condition for vitamin D insufficiency. The American Institute of Medicine and the European Food Safety Authority (EFSA) defined the same cut-off of vitamin D for adults and pregnant women fixing the value of sufficiency blood levels of 25(OH)D above 20 ng/mL (50 nmol/L) [55,62]. Nevertheless, some researchers debated the possibility that pregnancy would need higher concentrations of vitamin D [24].

Insufficiency and deficiency for vitamin D are common among pregnant women worldwide, due to foetal growth, low exposure to sunlight, and low dietary intake [63]. A comprehensive analysis of existing studies conducted on pregnant and lactating women worldwide showed low serum vitamin D levels especially in Asia, with the highest prevalence of deficiency in Kuwait (38–41%), Pakistan (45%), Turkey (50%), and India (60%) [64]. In Europe, also the Netherlands and Belgium had a relevant percentage of deficiency (23% and 12%, respectively); low vitamin D status was also found in some high-income countries such as Spain (20%), Canada (24%), America (33%), UK (35%), Netherlands (44%), Belgium (45%), and Germany (77%), suggesting that there is a potential global insufficient vitamin D consumption resulting in a common deficiency especially during pregnancy [64].

Deficiency criteria may depend also on methods used because of the wide availability of different analytical techniques for the detection of markers for vitamin D. Even the preparations (i.e., solvent extraction or the sample pre-treatment) adopted in different laboratories for the same protocol may affect results [65,66]. Currently, there is a massive use of immunoassays methods thanks to automation, however, there is the need of a consensus that harmonizes the cut-offs of various techniques used such as high-performance liquid chromatography (HPLC), liquid chromatography-tandem mass spectrometry (LC-MS/MS), chemiluminescence immunoassays (CLIA), enzyme-linked immunosorbent assays (ELISA), competitive protein binding assays (CPBA), and radioimmunoassays (RIA) [67,68,69,70]. It seems that other methods with respect to HPLC or LC-MS/MS underestimate serum levels of vitamin D [71]. However, all methods could suffer from the lack of a common standard and differences of methods applied among different laboratories.

## 4. Evidences of Relation between Vitamin D and GDM

### 4.1. Observational Studies

Table 1 summarize the main data from meta-analyses of observational studies: Poel, et al. in a systematic review and meta-analysis of seven studies including 2146 participants found an association between 25(OH)D blood concentration and GDM (Odds Ratio (OR) 1.61, 95% Confidence Interval (CI): 1.19–2.17) [72]. Aghajafari, et al. have conducted a meta-analysis of 31 observational studies and have found a correlation between low levels of 25(OH)D and risk for GDM (OR 1.49, 95% CI: 1.18–1.89) [24]. Similar, Wei, et al. have conducted a meta-analysis of 12 studies with 5,615 patients and have found a moderate correlation between 25(OH)D concentrations below 50 nmol/L of pregnant women and an increased risk of GDM (OR 1.38, 95% CI: 1.12–1.70) [73]. More recently, Zhang and colleagues found similar conclusions after a meta-analysis of 20 studies conducted in Europe, Australia, North America, and Asia with different study design including cross-sectional, case-control, nested case-control, and cohort studies including 9209 participants [74]. Quality assessment indicated a homogeneous high-quality score among studies and, even if seven different criteria for GDM diagnosis and five different assay techniques for serum vitamin D detection were used, an overall association between vitamin D deficiency and risk of GDM was found (OR 1.53, 95% CI: 1.33–1.75) with 4.93 nmol/L decreased levels of 25(OH)D in case of GDM. There was a high diversity of participant characteristics with a prevalence of vitamin D deficiency from 3.1% to 94.7% with a 25(OH)D concentration ranged from 16.5 to 97.0 nmol/L with a median of 49.30 nmol/L. Unfortunately, not all studies provided analysis adjusted for confounding factors such as skin tone and BMI, thus heterogeneity was high (*I^2^* = 61%). Lu, et al. in 2016 published a wider and more updated meta-analysis of 20 observational studies with 16,515 individuals and found an association between vitamin D insufficiency and GDM risk (RR 1.45, 95% CI: 1.15–1.83) [75]. Even in this meta-analysis the geographical origin was heterogeneous, accounting for studies from Australia, UK, Iran, Spain, Turkey, China, Korea, India, America, and Canada that may contribute to the differences in the subgroup analysis associations together with differences in adjusted models, study design, and assessment of vitamin D (laboratory techniques used and cut-offs). Again, heterogeneity was high (*I^2^* = 66%). Characteristics of the studies were crucial to notice the association: In fact, vitamin D insufficiency was associated with GDM in studies using HPLC-MS assay but not when radioimmunoassay was used. Similarly, in a systematic review and meta-analysis of 26 observational studies by Amraei and coworkers, a relation between vitamin D deficiency and risk of GDM (OR 1.18, 95% CI: 1.01–1.35) was shown [76]. Serum 25(OH)D in GDM patients were lower than controls with no significant association with population nationality of the studies. However, study design (cross-sectional, prospective, nested case-control, retrospective, or prospective cohort), in the subgroup analysis showed a variable association. Interestingly, there was limited evidence of heterogeneity among the reviewed studies (*I^2^* = 8.1%, *p* = 0.346). Standardized mean difference (SMD) between vitamin D levels in women with GDM and those with normal glucose tolerance was −0.26 (95% CI: From −0.39 to −0.14) with high heterogeneity (*I^2^* = 68.8%). Limitations of this study were the heterogeneity of GDM criteria, vitamin D cut-offs, and the lack of important information in some studies such as skin color, socio-economic status, weight gain in pregnancy, exposure to sunlight, and vitamin D intakes. The association between increased risk of GDM and vitamin D insufficiency was also confirmed by Hu at al. in a meta-analysis of 29 observational studies that included 28,982 participants (OR 1.39, 95% CI: 1.20–1.60) with significant high heterogeneity (*I^2^* = 50.2%) [77]. Moreover, differences of 25(OH)D levels between patients with GDM and patients without any issue of glucose tolerance was significant but with high heterogeneity (−4.79 nmol/L, 95% CI: From −6.43 to −3.15, *I^2^*= 65.0%). 72% of studies were conducted in developed countries but there was no homogeneity for GDM and 25(OH)D cut-offs, as can be expected. As for other meta-analyses, also in this work the adjustments for confounding factors such as BMI, ethnicity, season, gestational age, physical activity, pregnancy weight gain, parity, vitamin D intake, alcohol, and socioeconomic status. were in different combinations. Interestingly, stratification of the studies in the meta-analysis showed that the correlation between vitamin D insufficiency and risk of GDM was significant (i) for developed countries rather than for the developing ones; (ii) for GDM cut-offs of ADA, ADPS, and WHO but not for those from other organizations; and (iii) in case of significant difference of 25(OH)D levels between GDM and control but not when difference was not significant.

### 4.2. Clinical Trials

Vitamin D supplementation for GDM seems to ameliorate different metabolic markers including blood glucose levels, insulin resistance and inflammatory biomarkers [78,79,80]. Supplementation with 50,000 International Units (IU) twice monthly has been shown to improve insulin resistance significantly [79], while 5000 IU daily has failed to improve glucose levels in another trial [81]. Table 2 summarises the results of selected meta-analyses of Randomized Controlled Trials (RCTs). A meta-analysis in 2012 showed the protective efficacy of vitamin D supplementation (800–1000 IU daily) for mother and child’s health outcomes [82]. Yap at al. found no change in glucose concentration among Australian first-trimester pregnant patients with a high dosage intervention of vitamin D supplementation [81]. However, the study was not adequately designed to explore the incidence of GDM and it was based on a low-risk population for vitamin D deficiency. In fact, in a randomized controlled trial, Sablok and co-workers showed about half of the GDM prevalence among vitamin D treated group with respect to non-intervention in 200 Indian pregnant women [83]. Unfortunately, in two recent meta-analyses about oral supplementation of vitamin D, there was no association with GDM, even if vitamin D assumption led to higher levels of 25(OH)D compared to control [84,85]. Pérez-Lòpez, et al. published a systematic review and meta-analysis of 13 RCTs (2299 participants) on the effect of vitamin D in pregnancy [85]: Vitamin D supplementation was associated with increased circulating 25(OH)D levels (mean difference 66.5 nmol/L, 95% CI: 66.2–66.7), but it did not influence GDM and other maternal outcomes including pre-eclampsia, short for gestational age, caesarean section, and low birth weight. Besides the moderate heterogeneity (possibly due to different geographical areas investigated, dosage, duration, type of supplementation, or method of measurement), some methodological issues should be taken into account, including intervention in the second semester of pregnancy and lack of correction for some confounding factors, such as nutritional aspects that may be crucial when considering developing countries. Palacios and co-workers conducted an updated meta-analysis about the supplementation of vitamin D during pregnancy that included 15 RCT involving 2,833 women regarding multiple outcomes in pregnancy [84]: Supplementation with vitamin D raised serum 25(OH)D levels compared to placebo (mean difference 54.7 nmol/L, 95% CI: 36.6–72.9): Coherently with the work of Pérez-Lòpez, the biomarker for vitamin D was affected by the intervention but no difference in the risk of GDM was found. However, studies that explored the risk of GDM were only two with low quality, thus the lack of association was not definitive. In a Cochrane review of 15 randomized and quasi-randomized trials including a total of 2833 women on vitamin D efficacy on pregnant outcomes, only three studies investigated GDM with no association, but these trials were the same described above by Palacios [86]. The rise of circulating 25(OH)D after supplementation, particularly on a daily basis, is confirmed, but with a high heterogeneous response. Data on the safety of supplementation was lacking in all studies. In 2017, Akbari and co-workers conducted a systematic review and meta-analysis of 11 RCT on the effects of vitamin D supplementation on glucose metabolism and lipid profiles in GDM patients (187 subjects and 184 controls) and found a significant effect of vitamin D on metabolic homeostasis models [87]: Researchers observed an improvement of homeostatic model assessment of insulin resistance (HOMA-IR) (SMD −0.66, 95% CI: From −1.14 to −0.18) and quantitative insulin sensitivity check index (QUICKI) (SMD 0.73, 95% CI: From 0.26 to 1.20), despite supplementation resulted in a worsening of the homeostatic model assessment β-cells function (HOMA-B) (SMD −0.52, 95% CI: From −0.79 to −0.25). Fasting plasma glucose, insulin, and HbA1c were not significantly affected. High heterogeneity was reported, possibly depending on study duration and participants’ characteristics and, more importantly, on vitamin D dosage.

## 5. Limitations of Current Evidence and Future Perspectives

Despite the summary results of observational studies seem convincing, their relevance in the evidence hierarchy and the frequent presence of heterogeneity (probably due to ethnicity, geographical characteristics, skin tone, gestational age, cut-offs, and laboratory methods used) makes difficult the transfer this knowledge into clinical practice. Without considering the evaluation of the quality of the meta-analyses included in this review (which is out of the scope of the present article), there is a gap between observational and intervention studies investigating the role of vitamin D in preventing GDM or ameliorating its clinical presentation. This is often observed in nutritional epidemiology, and it is currently the topic of a recent diatribe, which will not be discussed in this article neither. However, it is noteworthy to discuss the meaning of the findings here reviewed: A disagreement in findings between observational studies measuring vitamin D levels in association with occurrence of GDM and studies evaluating the efficacy of vitamin D supplementation (with no univocal methodology across studies included in the meta-analysis) in decreasing the risk of GDM is not necessarily equivalent to ambivalent results. Specifically, the observational studies assessed a clinical condition occurring following physiological processes (i.e., dietary consumption of vitamin D-rich foods, and sun exposure) while the RCTs showed the effect of dietary supplementation, which actually differ from the previous condition.

The highest level of evidence for efficacy of supplementation could be provided by the results of randomized, double-blind, placebo-controlled clinical trials. However, meta-analyses of clinical trials may still be influenced by potential confounding factors that ultimately affect the conclusions derived from these studies [88,89]. For instance, the gestational age of intervention can be crucial for restoring a situation of repletion. In fact, while 25(OH)D blood levels seem to promptly respond to supplementation, peripheral cellular deficiency may require more time, and intervention studies designed during the late gestational age may not consider sufficient time to restore physiological conditions useful for the prevention of dysfunctions [90]. The selection of factors to be controlled in the experimental setting is of great importance since it is well known that some aspects such as reduced physical activity, BMI, and high mother age are risk factors for both GDM and vitamin D deficiency [91,92]. Other factors strictly related to vitamin D, such as endogenous biosynthesis, type and dosage of vitamin D (including matrix used in the supplement and other substances), diet, etc, should ideally be taken into account when planning a trial. Dose-response studies can be crucial to design efficient intervention [93]. Dosage and duration of supplementation could be critical to establishing a relationship between intervention and outcomes. In fact, even large *boli* are rapidly absorbed and undetectable, while during the supplementation vitamin D levels reach a plateau after three to four months of continuous provision [94,95]. In a relatively short window like pregnancy, this aspect could be crucial. The specific vitamin D isoform used could play a role in the conflicting results. Though vitamin D_2_ and vitamin D_3_ follow the same metabolic fate after absorption, the latter isoform seems to provoke a more marked increase of 25(OH)D blood levels after supplementation [23,96]. The same dosage of vitamin D_3_ rises two-fold the serum levels of 25(OH)D in respect to vitamin D_2_ supplement [23,96]. This phenomenon occurs both in the short-term and in the long-term administration [97,98,99]. The greater efficiency of the vitamin D_3_ form seems to depend on the more marked affinity towards the vitamin D binding protein [100,101,102]. The current state of the art suggests that, although the D_3_ form could be a preferential substrate for the hepatic hydroxylation, and therefore in raising the circulating levels more efficiently in the case of high bolus doses, at physiological daily concentrations there do not seem to be any differences between the two isoforms [103].

## 6. Conclusions

There is a need for larger well-designed RCTs that evaluate interventions together with the evaluation of numerous confounding factors. However, first of all, it would be useful to define the cut-offs, detection methods, and dosages for a more homogeneous analysis of such complex phenomenon as the GDM. The choice of the population is decisive, therefore, to obtain a clear and convincing result. Populations replete for vitamin D and with an effective program of preventive supplementation may not respond to vitamin supplementation while other factors such as smoking, weight, and gestational age, may mask the outcomes of the intervention. Supplementation should be calibrated on the variation of circulating 25(OH)D and not on the intervention dosage.

Because GDM establishes in a very early stage of pregnancy, even the first trimester could be too late for interventional supplementation and periconceptional period could be the most suitable for interventions. The role of PTH in GDM must be elucidated and therefore intervention studies should be able to discriminate from the possible phenomenon of PTH suppression by vitamin D during pregnancy supplementation. Understanding the adaptive mechanisms in pregnancy and also the bioavailability of vitamin D in its isoforms and in relation to the expression of vitamin D binding protein and receptor could increase the interpretative power in clinical trials. Taking into account the diffused vitamin D insufficiency among pregnant women, vitamin D can be a good candidate for the prevention and management of GDM with no relevant risks in case of intervention protocols at physiological dosages. However, it must be integrated into broader approaches that take into consideration other determinants that can effectively influence the multifactorial nature of the disease.

## Figures and Tables

**Table 1:** Association between insufficient vitamin D and risk of Gestational Diabetes Mellitus in selected meta-analyses of observational studies.

References	No. Studies	No. Participants	Risk (95% CI)	*I^2^*
Poel, et al. 2012 [72]	7	2146	OR 1.61 (1.19–2.17)	5.8%
Aghajafari, et al. 2013 [25]	31	4112	OR 1.49 (1.18–1.89)	0.0%
Wei, et al. 2013 [73]	12	5615	OR 1.38 (1.12–1.70)	0.0%
Zhang, et al. 2015 [74]	20	9209	OR 1.53 (1.33–1.75)	16.2%
Lu, et al. 2016 [75]	20	16,515	RR 1.45 (1.15–1.83)	66.6%
Amraei, et al. 2018 [76]	26	20,503	OR 1.18 (1.01–1.35)	8.1%
Hu, et al. 2018 [77]	29	28,982	OR 1.39 (1.20–1.60)	50.2%

OR: Odds Ratio, RR: Risk Ratio, I^2^: Heterogeneity.

**Table 2:** Association between intervention with vitamin D and GDM outcomes in meta-analyses of RCTs.

References	No. Studies	No. Participant	Outcomes	*I^2^*
Pérez-Lòpez, et al. 2015 [85]	13	2299	Circulating 25-OHD: (MD 66.46, CI 95% 66.22 to 66.71)	100.0%
			Preeclampsia: (RR 0.88, CI 95% 0.51 to 1.52)	24.0%
			Gestational diabetes: (RR 1.05, CI 95% 0.60 to 1.84)	0.0%
			Small for gestational age: (RR 0.78, CI 95% 0.50 to 1.21)	15%
			Low birth weight: (RR 0.72, CI 95% 0.44 to 1.16)	0.0%
			Preterm birth: (RR 1.26, CI 95% 0.60 to 2.63)	0.0%
			Birth weight: (MD 107.60, CI 95% 59.86 to 155.33)	0.0%
			Birth length: (MD 0.30, CI 95% 0.19 to 0.41)	84.0%
			Cesarean section: (RR 0.94, CI 95% 0.78 to 1.13)	0.0%
Palacios, et al. 2016 [84]	7	868	Serum 25-OHD: (MD 54.73, CI 95% 23.76 to 70.39)	99.0%
			Preeclampsia: (RR 0.52, CI 95% 0.25 to 1.05)	15.5%
			Gestational diabetes: (RR 0.43, CI 95% 0.05 to 3.45)	NR
de-Regil, et al. 2016 [86]	3	273	Circulating 25-OHD: (MD 47.08, CI 95% 36.60 to 72.86)	98.0%
			Low birth weight: (RR 0.48, CI 95% 0.23 to 1.01)	53.0%
			Birth length: (MD 0.97, CI 95% −0.41 to 2.34)	84.0%
			Head circumference: (MD 0.43, CI 95% 0.06 to 0.79)	50.0%
			Birth weight: (MD 39.55, CI 95% −240.68 to 319.78)	96.0%
Akbari, et al. 2017 [87]	11	371	HOMA-IR: (SMD −0.66, CI 95% −1.14 to -0.18)	57.4%
			QUICKI: (SMD 0.73, CI 95% 0.26 to 1.20)	64.9%
			HOMA-B: (SMD −0.52, CI 95% −0.79 to −0.25)	0.0%

OR: Odds Ratio, RR: Risk Ratio, MD: Mean Difference, SMD: Standardized Mean Difference, I^2^: Heterogeneity, NS: Not Significant.
